# Investigation of Negative Bias Temperature Instability Effect in Nano PDSOI PMOSFET

**DOI:** 10.3390/mi13050808

**Published:** 2022-05-23

**Authors:** Yafang Yang, Hongxia Liu, Kun Yang, Zihou Gao, Zixu Liu

**Affiliations:** Key Laboratory for Wide Band Gap Semiconductor Materials and Devices of Education, School of Microelectronics, Xidian University, Xi’an 710071, China; yfyang_xd@163.com (Y.Y.); kuny2019@163.com (K.Y.); gaozih_xd@163.com (Z.G.); zxliu0026@163.com (Z.L.)

**Keywords:** negative bias temperature instability, partially depleted silicon-on-insulator, threshold voltage shift, NBTI lifetime, floating-body

## Abstract

The Negative Bias Temperature Instability (NBTI) effect of partially depleted silicon-on-insulator (PDSOI) PMOSFET based on 130 nm is investigated. First, the effect of NBTI on the IV characteristics and parameter degradation of T-Gate PDSOI PMOSFET was investigated by accelerated stress tests. The results show that NBTI leads to a threshold voltage negative shift, saturate drain current reduction and transconductance degradation of the PMOSFET. Next, the relationship between the threshold voltage shift and stress time, gate bias and temperature, and the channel length is investigated, and the NBTI lifetime prediction model is established. The results show that the NBTI lifetime of a 130 nm T-Gate PDSOI PMOSFET is approximately 18.7 years under the stress of VG = −1.2 V and T = 125 °C. Finally, the effect of the floating-body effect on NBTI of PDSOI PMOSFET is investigated. It is found that the NBTI degradation of T-Gate SOI devices is greater than that of the floating-body SOI devices, which indicates that the floating-body effect suppresses the NBTI degradation of SOI devices.

## 1. Introduction

**Figure 1:**
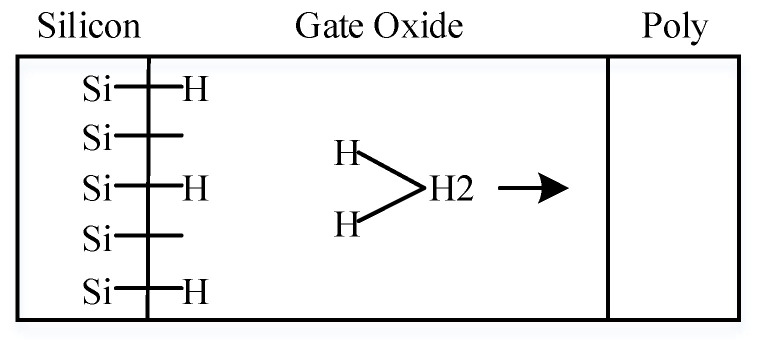
Schematic illustration of the R-D model to interpret interface trap generation.

With the shrinking size of the integrated circuit and the thinning gate oxide thickness of MOSFETs, negative bias temperature instability (NBTI) has become a major reliability issue in modern CMOS technology [1]. It mainly describes the performance degradation of the PMOSFET when operating at negative gate bias and high temperature, which is mainly manifested as the threshold voltage shift, transconductance drop, and saturate current decrease of the PMOSFET due to the interface trap at Si/SiO_2_ and the trap charge generated in the gate oxide [2]. Researchers have proposed many models to interpret the degradation mechanism of NBTI, among which the reaction-diffusion (R-D) model has been widely applied [3]. The R-D model assumes that when a bias is applied to the gate, a reaction related to the electric field will occur at the Si/SiO_2_ interface, and the passivated Si-H bonds will be broken, resulting in interface traps, as shown in Figure 1. Meanwhile, the high temperature will weaken the existing Si-H bonds, so it will also aggravate NBTI [4].

Recently, silicon-on-insulator (SOI) technology has been widely used because of its main advantages, including latch-up immunity and high speed [5]. Whereas the NBTI effect has a more severe impact on the reliability of SOI devices, and it is found that the NBTI degradation of SOI devices is greater than that of CMOS devices [6]. Compared with CMOS devices, SOI devices will produce a self-heating effect under the action of NBTI stress due to the existence of buried oxide, resulting in the increase of device channel temperature. According to the degradation mechanism of the NBTI effect, higher temperature will make the NBTI degradation of PMOSFET more serious. Moreover, it is found that most of the current research on NBTI lifetime prediction is based on CMOS devices [7], while the research on the NBTI lifetime of SOI devices is very lacking, and most of them only involve the NBTI failure mechanism and electrical performance degradation, and there is no complete NBTI lifetime prediction model [8,9,10]. Therefore, the basic research on NBTI and the establishment of the NBTI lifetime model in this paper are of great significance.

In addition, the research object of this paper is mainly partially depleted (PD) SOI devices. Due to their own structural characteristics, PDSOI devices have the floating-body effect, which will have a negative impact on the device characteristics [11]. The floating-body effect can be suppressed by building body contact, which mainly includes T-Gate and H-Gate. In order to further study the NBTI of SOI devices, the influence of the floating-body effect on NBTI is studied in this paper.

In this paper, the transfer characteristics and sensitive parameters degradation due to NBTI of a 130 nm PDSOI technology are investigated. The stress time, electric field, temperature, and channel length dependence of NBTI characterized by parameter shifts of PMOSFET are studied, and the transistor lifetime is evaluated. The following section will elaborate on the devices used and experimental details. The next section discusses the experimental results obtained by the NBTI experiments, estimates the lifetime of NBTI, and investigates the effect of floating body on NBTI.

## 2. Materials and Methods

**Figure 2:**
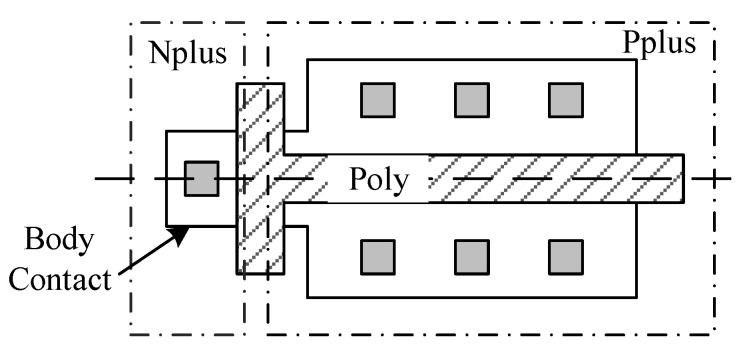
Layout of PMOS transistor used in our study.

All devices used in the experiments were fabricated based on the 130 nm PDSOI process. The top Si film thickness is 100 nm, and the buried oxide thickness is 145 nm. The Core and I/O devices are selected as samples in our experiments, and both devices are described in Table 1. As shown in Figure 2, a T-Gate is used for body contact to suppress the floating-body effect.

The NBTI stress experiments were conducted by an Agilent B1500 semiconductor parameter analyzer [12] using the quasi-DC Stress-Measure-Stress (SMS) technique [7]. NBTI measurement includes applying voltage stress higher than the operating voltage to the gate at a high temperature to accelerate degradation. The source, drain, and substrate contacts were grounded in this experiment [13]. The wafers were subjected to different stress temperatures of 100, 125, and 150 °C, and the applied stress biases at the gate were −1.8, −2.0, and −2.2 V [14]. The total stress time was 3000 s, with periodic interruptions. The following device parameters were measured to monitor device degradation under stress: V_th_, I_ds_, and g_mmax_. A complete I_d_~V_g_ curve was measured before and after the stress with V_d_ = −0.1 V, and V_th_ is the threshold voltage extracted through the maximum transconductance method. I_ds_ was extracted from the I_d_~V_g_ curve, and its value is equal to the corresponding I_d_ value when V_g_ = −1.2 V in the I_d_~V_g_ curve. g_m_ was obtained by differentiating the I_d_~V_g_ curve, and g_mmax_ is the maximum value in g_m_.

In addition, the Sentaurus TCAD simulation tool was used to analyze the internal mechanism of the degradation of electrical characteristics of PDSOI PMOS devices before and after NBT stress [15]. The models used in the simulation process mainly include the trap degradation model, the mobility degradation model, and the recombination model [16].

## 3. Results and Discussion

### 3.1. NBTI Degradation of I–V Characteristic

**Figure 3:**
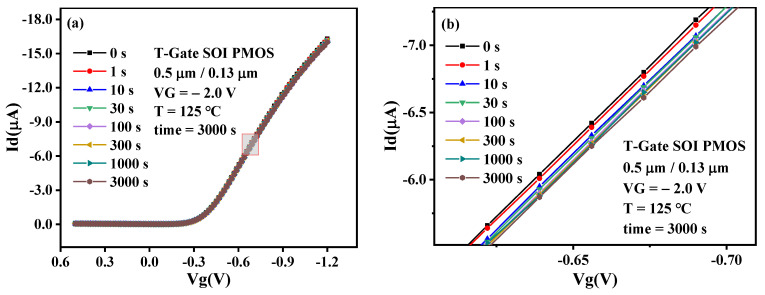
Transfer characteristics of PMOS before and after NBT stress. (**a**) Complete, (**b**) Enlarged.

**Figure 4:**
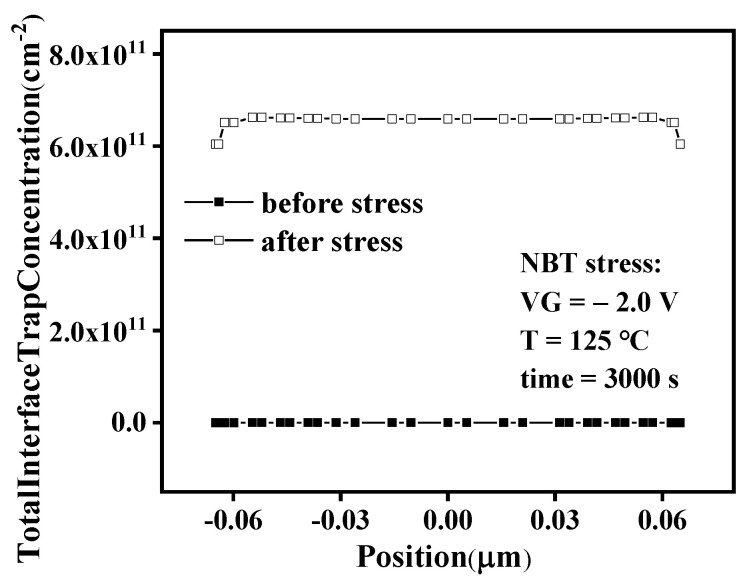
Distribution of interface trap concentration in PMOSFET.

Figure 3 shows the transfer characteristics of a Core PMOS with W/L = 0.5 μm/0.13 μm before and after stress. T = 125 °C and VG = −2.0 V were selected as the main stress conditions to prevent the SOI device’s failure due to excessive temperature or excessive gate voltage during the test. It can be observed that the negative shift of threshold voltage after NBT stress. The threshold voltage is changed from −0.32166V to −0.33182 V, and the shift is about 10.16 mV. It is caused by the interface trap at Si/SiO_2_ and the trap charge generated in the gate oxide [2]. This paper verifies this phenomenon through TCAD simulation; the simulation results are shown in Figure 4. It can be observed from the figure that the concentration of the interface trap at Si/SiO_2_ after NBT stress is significantly increased, and the increase in trap charge will cause a negative shift in the threshold voltage. In order to evaluate the reliability of MOSFET, the threshold voltage shift (Δ*V_th_*) is often used as the evaluation standard. Δ*V_th_* can be expressed as follows:

(1) $$\Delta V_{th}=\frac{-q(\Delta N_{ot}+\Delta N_{it})}{C_{ox}}=\frac{-q(\Delta N_{ot}+\Delta N_{it})t_{ox}}{\varepsilon_{ox}}$$

where *q* is the electron charge, *C_ox_* is the gate oxide capacitance, *t_ox_* is the gate oxide thickness, *ε_ox_* is the permittivity of the oxide, and Δ*N_ot_* and Δ*N_it_* are the density of stress-induced oxide trapped charge and interface trap at Si/SiO_2_, respectively.

Figure 4 shows the interface trap concentration at Si/SiO_2_ of the SOI PMOS device before and after stress. It can be observed from the figure that the concentration of interface traps at Si/SiO_2_ increases significantly after NBT stress, which is because under the action of NBT stress, the Si-H bond is broken, and, finally, the electrically active interface traps are formed, resulting in the trap concentration at the interface increasing.

**Figure 5:**
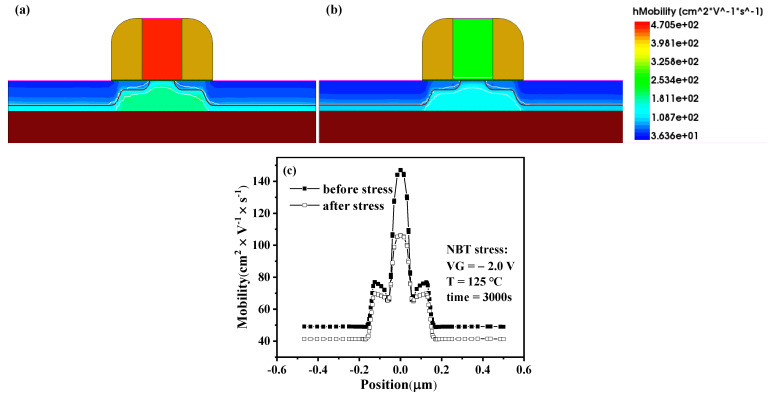
Distribution of carrier mobility in PMOSFET. (**a**) Before stress, (**b**) After stress, (**c**) Compared.

In addition, the reduction of the drain current at V_g_ = −1.2 V after NBT stress can be observed in Figure 3, and the drain current varies from 16.3 μA to 15.8 μA, the shift is about 0.5 μA. The drain current reduction is caused by the threshold voltage and mobility. The interface trap concentration at Si/SiO_2_ increased significantly after the stress, causing a chance of scattering of the device [17], and the internal carrier mobility decreased, resulting in the reduction of the drain current. This paper verifies this phenomenon through TCAD simulation; the results are shown in Figure 5. It is observed that the device channel carrier mobility declines after NBT stress. This is because under the conditions of high temperature and negative gate voltage, the internal lattice collision of the device is intensified, and the scattering probability increases, which leads to the deterioration of the carrier mobility.

**Figure 6:**
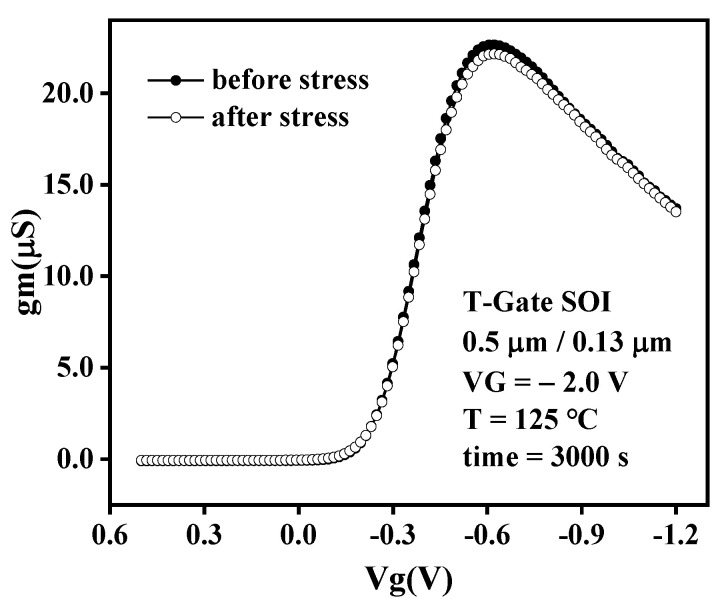
Transconductance characteristics of Core PMOS before and after NBTI stress.

Transconductance is the embodiment of the control ability of gate voltage to drain current. The higher the transconductance, the better the high-frequency response characteristics of the device. Figure 6 shows the transconductance characteristics of PMOSFET before and after stress. It can be observed that the transconductance decreases, and the maximum transconductance is a more negative gate voltage shift after NBT stress. As shown in Figure 6, the maximum transconductance shifts from 22.6 μS corresponding to V_g_ = −0.571 V to 22.1 μS corresponding to V_g_ = −0.662 V. Since the transconductance is proportional to the mobility in the linear region, and NBT stress leads to the decrease of carrier mobility, the device transconductance decreases after NBT stress.

**Figure 7:**
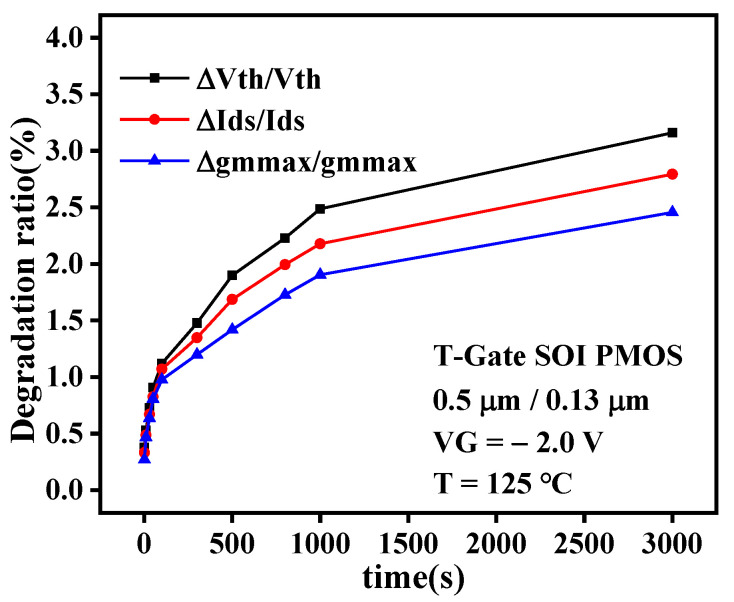
Degradation comparison of sensitive parameters after NBT stress.

It can be seen from the above analysis that the NBTI effect mainly leads to the degradation of electrically sensitive parameters, such as threshold voltage, saturated drain current, and maximum transconductance, of PMOS devices. Figure 7 shows the relationship between the shift of the sensitive parameter and the stress time. It can be observed from the figure that under the same stress condition, the degradation trend of threshold voltage, drain current, and transconductance is the same, which is shown as follows: with the increase of stress time, the degradation of sensitive parameters increases gradually. However, their degradation amounts are different, with the threshold voltage degradation being the largest. Therefore, the degradation of the threshold voltage is the main in the later research process; that is, the NBTI lifetime prediction model of SOI PMOS devices is established based on the threshold voltage shift.

### 3.2. Construction of NBTI Lifetime Prediction Model

The study found that the NBTI degeneration of the device can be represented by the following empirical models [18],

(2) $$\Delta V_{th}\propto {\left(\frac{1}{L}\right)}^{p}\times \exp(-\frac{E_{a}}{KT})\times \exp(-\frac{C}{\left|VG\right|})\times t^{n}$$

where *n* is the time exponent factor, *p* is the channel length influence factor, *E_a_* is the activation energy, and *C* is the electric field acceleration factor. These parameters can be extracted from the experimental results.

#### 3.2.1. Stress Time Dependence of NBTI Degradation

**Figure 8:**
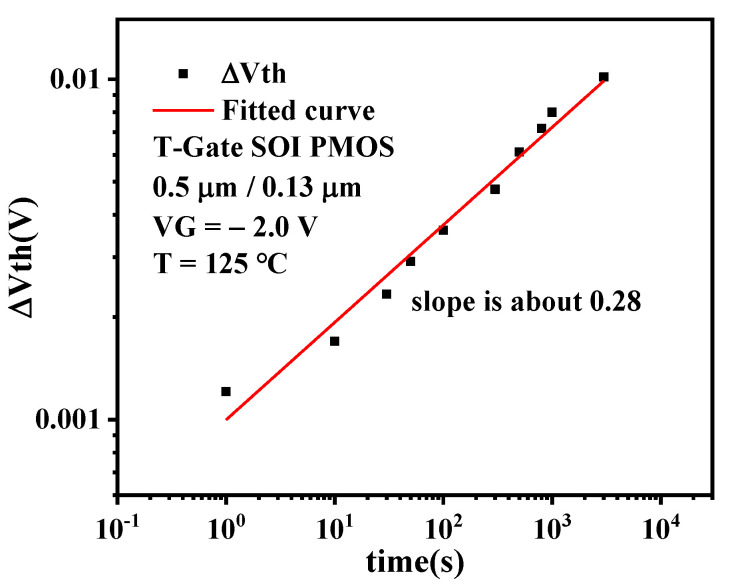
Δ*V_th_* degradation dependence on the stress time.

Figure 8 presents Δ*V_th_* degradation dependence on the stress time. It can be observed that the threshold voltage shift increases with the increase of NBT stress time and is approximate to a straight line in double logarithmic coordinates. That is, the relationship between the threshold voltage and stress time conforms to the power exponent and the time exponent factor *n* = 0.28 [19].

#### 3.2.2. Gate Bias Dependence of NBTI Degradation

**Figure 9:**
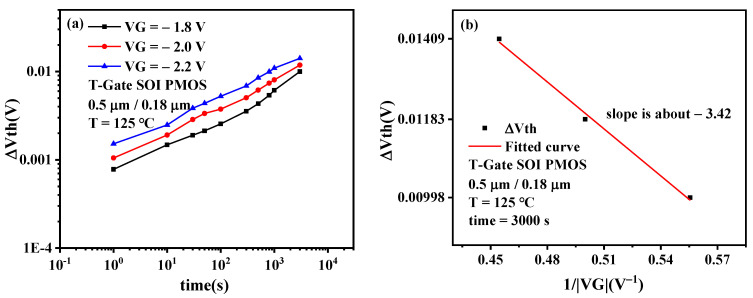
(**a**)Time evolution of Δ*V_th_* degradation under different gate biases. (**b**) Δ*V_th_* degradation dependence on the stress bias.

Figure 9a presents the time evolution of Δ*V_th_* degradation under different gate biases. It can be observed from the figure that under different gate bias stress, the threshold voltage shift of SOI PMOS device will increase with the increase of stress time after NBT stress, and the greater the gate bias stress, the more serious the damage degree of the device, which indicates that increasing the gate bias stress can accelerate the NBTI degradation of the PMOS device. The reason for this phenomenon is that when the thickness of the gate oxide of the SOI PMOS device remains unchanged, the increase of the stress gate voltage will lead to an increase in the longitudinal electric field intensity of the device. According to the degradation mechanism of the NBTI effect, the increase of gate electric field intensity will make the process of hole injection into the gate dielectric layer of PMOS devices easier, which will lead to more holes in the gate oxide. Holes can accelerate the reaction-diffusion process inside PMOS devices, which is reflected in the greatly increased fracture probability of the Si-H bond at the Si/SiO_2_ interface. This phenomenon leads to the greatly increased number of H material diffused into the gate oxide, which makes the NBTI degradation of PMOS devices worse.

Figure 9b presents Δ*V_th_* degradation dependence on the stress gate bias. It can be observed that there is a linear relationship between the threshold voltage degradation and the reciprocal of the gate bias in semi-logarithmic coordinates, which meets the empirical model-exp model. The electric field acceleration factor can be extracted from Figure 9b; that is, *C* = 3.42.

#### 3.2.3. Temperature Dependence of NBTI Degradation

**Figure 10:**
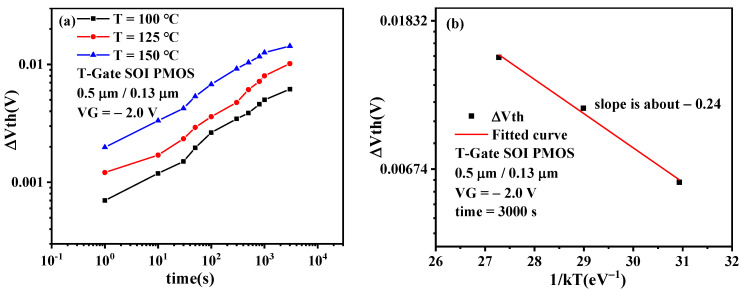
(**a**)Time evolution of Δ*V_th_* degradation under different temperatures. (**b**) Δ*V_th_* degradation dependence on the stress temperature.

Figure 10a presents the time evolution of Δ*V_th_* degradation under different temperatures. It can be observed from the figure that under different stress temperatures, the threshold voltage shift of the device under test after NBT stress increases with the increase of stress time, and the NBTI degradation of the device becomes more severe with the increase of temperature; that is, an increase in temperature will accelerate the NBTI degradation of SOI PMOS devices. The reason for this phenomenon is that high temperatures accelerate the fracture of Si-H bonds at the Si/SiO_2_ interface of PMOSFET, and high temperatures can accelerate the diffusion of by-product H produced in the R-D process into the gate oxide. These two factors lead to the intensification of NBTI degradation of PMOSFET [20].

Figure 10b presents the Δ*V_th_* degradation dependence on the stress temperature. It can be observed that there is a linear relationship between the threshold voltage shift and the reciprocal of the product of temperature and Boltzmann constant in semi-logarithmic coordinates; that is, the relationship between the threshold voltage shift and stress temperature conforms to the empirical model Arrhenius equation. From Figure 10b, the activation energy, *E_a_* is extracted to be 0.24 eV [21].

#### 3.2.4. Channel Length Dependence of NBTI Degradation

**Figure 11:**
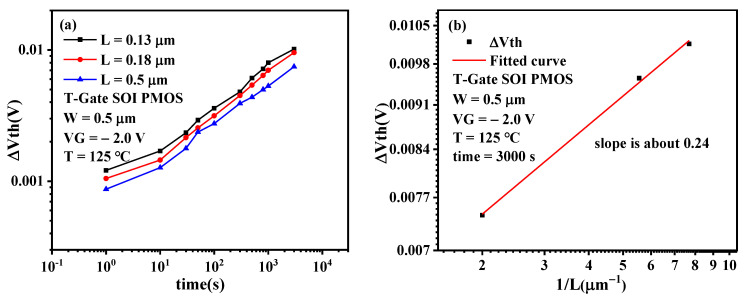
(**a**) Time evolution of Δ*V_th_* degradation under different channel lengths. (**b**)Δ*V_th_* degradation dependence on the channel length.

Figure 11a presents the time evolution of Δ*V_th_* degradation under different channel lengths. It can be observed from the figure that the threshold voltage degradation trend of PMOSFET under different channel lengths is similar, and with the decrease of channel length, the threshold voltage shift increases; that is, the degradation of NBTI becomes worse [22].

Figure 11b presents Δ*V_th_* degradation dependence on the channel length. It can be observed that there is a linear relationship between the threshold voltage shift and the reciprocal of the channel length in the double logarithmic coordinate; that is, there is a power–law relationship between the threshold voltage shift and the reciprocal of the channel length. From Figure 11b, the channel length exponent factor, *p* is extracted to be 0.24.

It can be seen from the above discussion that the time exponent factor is *n* = 0.28, the activation energy is *E_a_* = 0.24, the electric field acceleration factor is *C* = 3.42, and the channel length exponent factor is *p* = 0.24. Therefore, the empirical model can be transformed into:

(3) $$\Delta V_{th}=A\times {\left(\frac{1}{L}\right)}^{0.24}\times \exp(-\frac{0.24}{KT})\times \exp(-\frac{3.42}{\left|VG\right|})\times t^{0.28}$$

where the proportional constant *A* is related to the specific process. The lifetime of PMOSFET can be measured under constant high temperatures and gate voltage stress, and then the value of *A* can be determined.

The NBTI lifetime of PMOSFET can be defined as the time when the threshold voltage shifts by 100 mV when the temperature is 125 °C and the gate is at normal working voltage. It is deduced that the NBTI lifetime of 130 nm TB PDSOI PMOS device is about 18.7 years.

### 3.3. Influence of Floating Body on NBTI of SOI Devices

**Figure 12:**
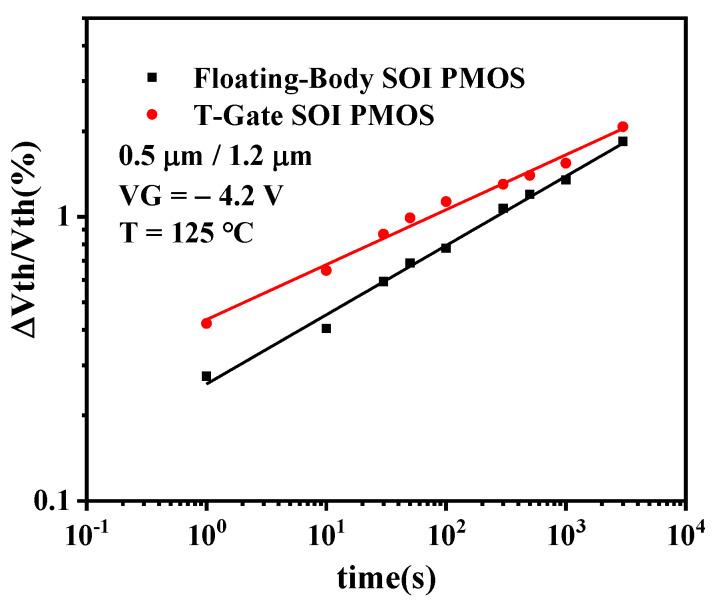
Comparison of NBTI degradation between floating-body and T-Gate SOI PMOSFET.

Figure 12 shows the time dependence of the threshold voltage shift of the floating-body and the T-Gate SOI PMOSFET after NBT stress, from which it can be seen that the NBTI degradation trend of the floating-body SOI device is similar to that of the T-Gate SOI device, which shows that the threshold voltage degradation increases with the stress time and is linearly related to the stress time in double logarithmic coordinates. The time acceleration factor is about 0.25, and this phenomenon indicates that the presence of the floating body does not change the NBTI degradation mechanism of the PDSOI PMOSFET. It can also be observed that the degradation of the T-Gate SOI device is greater than that of floating-body SOI devices in a short stress time because, in floating-body SOI devices, tunneling electrons accumulate in the substrate, resulting in a lower potential in the body region, which further reduces the longitudinal gate oxide electric field. This results in a reduction in the number of holes in the channel inversion layer that form the interfacial state and oxide trap charges [23], leading to less NBTI degradation in floating-body SOI devices than in T-Gate SOI devices in a short stress time [24].

## 4. Conclusions

In this paper, the NBTI effect of PMOSFET from 130 nm PDSOI technology were investigated. First of all, the IV characteristics of 130 nm PDSOI PMOSFET under NBT stress and the degradation law of electrical parameters are analyzed through experimental tests. The test results show that NBTI leads to a negative shift of the PMOSFET threshold voltage, reduction of drain current in the linear region, and reduction of maximum transconductance. Then, the effects of temperature, gate bias, and channel length on the NBTI effect of SOI devices were investigated. It was found that the threshold voltage degradation is greater at high temperature, large gate bias, and small channel length. In addition, based on the experimental results, the activation energy, electric field acceleration factor, and time and channel length-related parameter factors were extracted to establish the NBTI lifetime prediction model for 130 nm PDSOI PMOSFETs. The NBTI lifetime of the device was inferred to be about 18.7 years under the stress of VG = −1.2 V and T = 125°C. Finally, the effect of the floating body on the NBTI of the PDSOI PMOSFET was investigated by comparing the NBTI degradation of the floating-body and T-Gate SOI devices. The results show that the NBTI degradation law of floating-body SOI devices is similar to that of T-Gate SOI devices, but the NBTI degradation of floating-body SOI devices is less than that of T-Gate SOI devices in a short stress time.

## Figures and Tables

**Table 1:** The two kinds of devices in the 130 nm PDSOI technology.

Device	Body Contact	Operating Voltage	Gate Oxide Thickness	Width-Length- Ratios (W/L)
Core	T-Gate	1.2 V	2 nm	0.5 μm/0.13 μm
				0.5 μm/0.18 μm
				0.5 μm/0.5 μm
I/O	T-Gate	3.3 V	7 nm	0.5 μm/1.2 μm
	Floating Body	3.3 V	7 nm	0.5 μm/1.2 μm
